# Association between head circumference at two years and second and fifth year cognition

**DOI:** 10.1186/s12887-021-02543-0

**Published:** 2021-02-11

**Authors:** Beena Koshy, Manikandan Srinivasan, Timiri Palani Murugan, Anuradha Bose, Pamela Christudoss, Venkata Raghava Mohan, Sushil John, Reeba Roshan, Gagandeep Kang

**Affiliations:** 1 Developmental Paediatrics Unit, Christian Medical College, Vellore, 632004 India; 2 Wellcome research Unit, Christian Medical College, Vellore, 632004 India; 3 Community Health, Christian Medical College, Vellore, 632004 India; 4 Clinical Biochemistry, Christian Medical College, Vellore, 632004 India; 5 Low Cost Effective Care Unit, Christian Medical College, Vellore, 632004 India

**Keywords:** Early childhood, Head circumference, Cognition

## Abstract

**Background:**

Head circumference (HC) measurement is routinely not performed in early childhood and there is conflicting information about its utility in literature. The current study analyses the association between HC at two years of age and cognition at two and five years of age.

**Methods:**

A community based birth-cohort recruited between 2010 and 2012 was followed up till five years of age in an urban slum in Vellore, India. Children were recruited at birth after informed parental consent by consecutive sampling using eligibility criteria of healthy new-born, singleton pregnancy and family’s availability in the study area during follow-up. HC measured at two years of age was used as the exposure variable to calculate association with cognition at both two and five years of age. Cognitive domain of Bayley scale of infant development was used at two years of age and Wechsler Preschool Primary Scales of Intelligence at five years.

**Results:**

Of the 251 enrolled children, 138 (55%) were girls and 71 (30%) belonged to lower socioeconomic status. At 2 years, 8.81% of children had HC < − 3SD. Compared to children with HC z-scores ≥ − 2 SD, those with measurements < − 3 SD had a lower cognition scores by − 2.21 [95% CI: − 3.87 - -0.56] at 2 years. Also, children with HC < − 3 SD at two years scored significantly lower scores in cognitive domains of verbal, − 7.35 [95% CI: − 11.78 - -2.92] and performance, − 7.07 [95% CI: − 11.77 - -2.36] intelligence at five years.

**Conclusions:**

This study showed that smaller HC at 2 years of age was negatively associated with cognition at both 2 and 5 years of age. Early childhood HC measurements can be utilised as a cheaper screening tool to identify children at risk in LMIC settings. Further studies can confirm these findings in diverse settings.

**Supplementary Information:**

The online version contains supplementary material available at 10.1186/s12887-021-02543-0.

## Introduction

Children growing up in vulnerable backgrounds including low and middle income country (LMIC) settings are at risk of growth and development faltering, necessitating their close monitoring [1, 2]. Anthropometric measurements in children including height, weight and head circumference (HC) measurements are used to gauge their maturational processes during childhood, and their close monitoring can identify early faltering necessitating appropriate and timely interventions [1]. Though height and weight are routinely measured in general paediatric clinics and immunisation centres, HC measurements are not consistently performed [3, 4]. HC measurement in early childhood/infancy reflects brain volume and growth in lieu of open cranial sutures and fontanelles; and can be a valuable tool to analyse brain growth and development in this age group [5, 6].

Studies evaluating the utility of HC measurements in childhood have produced mixed results. Many studies found routine HC measurement in typically developing children unnecessary, and suggested HC monitoring in instances where measurements were above + 2 SDs [5, 7, 8]. In children at risk, including those exposed to alcohol during prenatal period [9], born as a preterm [10, 11] and having very low birth weight [12], HC was shown to be related to cognition/development. Similar findings have been reported from typically developing children with evidences from a large multinational LMIC community based birth-cohort study [2], and from cohort studies in India [13], UK [14], Israel [15] and Uruguay [16]. An Indian birth-cohort study showed HC at birth was related to learning, memory and storage and visuospatial abilities around 10 years of age [13].

These diverging findings might be due to the timing of HC measurements and differing characteristics of the cohorts studied [5, 7–10, 12–14]. Relationship between HC and whole-brain volume is variable through human life with HC being an excellent predictor of brain volume in early childhood [17]. Once sutures and fontanelles close, HC remains almost unchanged, and does not reflect the later brain volume changes including age-dependent atrophy in later life.

With this background, we propose to assess the relationship between HC measured at two years and cognition at two and five years of age in a birth-cohort in south India. Two years is considered the culmination of the first 1000 days of life [18] and five years symbolises the end of the next 1000 days [19]; both signifying rapid brain growth and development. It is hypothesised that the two year HC measurement will be associated with both cognition at two and five years of age.

## Methods

### Settings and subjects

The current study was done as an independent sub-analysis of a large multinational prospective, longitudinal birth-cohort study in eight different countries across the world -‘The Etiology, Risk Factors and Interactions of Enteric Infections and Malnutrition and the Consequences for Child Health and Development (MAL-ED) Network’ [20]. The Indian study was conducted in a densely populated urban slum in Vellore, South India [21]. The population of 12,000 was covered prior to enrolment and pregnant women were identified by a door-to-door survey. Consenting mothers were invited by consecutive sampling to participate in the study immediately after birth, after an informed consent. The exclusion criteria included family’s existing plans to migrate out of the study site during the study period, multiple pregnancies, medical comorbidities in the index child, another child from the same family already registered in the study, and mothers unavailable to provide necessary consent. Trained field workers visited the recruited children twice a week till their second birthday for active disease surveillance and growth monitoring as per the MAL-ED protocol, with subsequent follow-ups. The initial birth-cohort recruitment in Vellore was between March 2010 and February 2012 and enrolled 251 new-borns. The original birth-cohort enrolment and subsequent follow-ups were approved by the Institutional Review Board and Ethics Committee of Christian Medical College, Vellore and children were recruited at each stage after informed parental consent.

### Measures

Anthropometric measurements were performed monthly for all children till two years of age. Length was measured using an infantometer to the nearest cm and weight using an electronic weighing scale to the nearest 10 g. HC was measured by a trained study nurse using a non-stretchable tape to the nearest 0.1 cm. Quality control was assured by periodic retraining and recalibration of machines as per the MAL-ED study protocol. We used Multicentre Growth Reference Study (MGRS) standards to calculate z-scores for height for age and the WHO growth standards for head circumference measurements respectively [22, 23].

### Bayley scales of infant and toddler development-III (BSID-III)

The Bayley Scales of Infant and Toddler Development-III (BSID-III) is a developmental measure and assesses motor, language, cognition and social skills development between 1 and 42 months of age [24]. The BSID-III was culturally adapted, translated, back-translated and piloted, prior to its administration; and testing was conducted by a single trained clinical psychologist. Around 10% of assessments were video-recorded and reviewed locally for quality control and 5–6% reviewed centrally by the central cognition team [25]. Cognition component of BSID-III measures exploration and manipulation of toys, concept formation, object relatedness and memory; and its raw scores at two years of age were utilised for current analysis.

### Wechsler preschool primary scales of intelligence-third edition (WPPSI-III)

The Wechsler Preschool Primary Scales of Intelligence – third edition (WPPSI-III) was used to assess cognition at five year of age [26]. This measure was adapted centrally for cultural appropriateness and was translated and piloted in local settings before being administered by a single trained psychologist. Verbal, performance and processing speed raw scores were obtained after administration for each child. For quality control, around 10% of assessments were recorded and reviewed centrally.

### The WAMI measure for socio-economic status

The WAMI measure is a composite measure of socio-economic status (SES) developed during the MAL-ED study, with components of access to improved Water and sanitation, Assets, Maternal education and total household Income [27]. A trained field worker visited the home and administered the translated and piloted WAMI measure at six-monthly intervals till 24 months. The final score calculated from these variables was converted to a standardized score ranging from 0 to 1.

### Raven’s progressive matrices

The Raven’s progressive matrices is a measure of non-verbal reasoning ability [28]. This test was administered by a trained psychologist to mothers to assess maternal cognition at 6–8 months of child’s age, as per the MAL-ED study protocol [25]. Raw scores were used for analyses.

### Blood collection and analysis

Blood samples were collected at 7, 15, 24 and 36 months of age in children. Ferritin and transferrin assays were done at 7, 15 and 24 months, and blood lead levels at 15, 24 and 36 months. The blood samples were immediately refrigerated using cold packs and transported to the research lab within two hours of blood collection. Ferritin and transferrin receptors were analysed using chemiluminescence immunoassay and immunoturbidimetry respectively, and blood lead level using Graphite Furnace Atomic Absorption Spectroscopy method in the Biochemistry department. Considering the influence of systemic inflammation on ferritin levels, we used a composite index based on both ferritin (F) and transferrin (T) levels to estimate the total body iron stores, with a positive value denoting surplus iron reserve [29].

$$ \mathrm{Body}\ \mathrm{Iron}\ \left(\mathrm{mg}/\mathrm{kg}\right)=\left(\frac{-\left(\log \left(\frac{R}{F} ratio\right)-2.8229\right)}{0.1207}\right) $$

### Data entry and analysis

Using a double entry database system managed by the Data Co-ordinating Centre of the MAL-ED study, information collected in paper forms were entered into electronic database [20]. Completed paper forms were validated by field supervisor before data entry.

### Statistical analysis

Categorical variables were expressed in percentages and continuous variables as mean (standard deviation) or median (range). Anthropometry measurements such as z-scores of HC, height-for-age, weight-for-age and weight-for-height were categorized into > = − 2SD, − 2 to − 3 SD and < − 3 SD. Using the 33rd percentile of WAMI scores as the cut-off, children were classified into low (<33rd percentile) and high (> = 33rd percentile) SES. Mean body iron and lead levels were calculated as average of measurements recorded at 7, 15 and 24; and, 15, 24 and 36 months; respectively. Demographic and anthropometric characteristics during enrolment, 2 and 5 years of the cohort were compared using Chi-squared statistics. Using simple linear regression, we modelled the effect of 24-month HC on cognition scores measured at 2 and 5 years, after adjusting for variables such as – sex, height-for-age scores at 24 months, mother’s cognition, SES, mean iron and lead levels. Blood lead levels, body iron and height for age z-scores during the early childhood were associated with cognition at 2 and 5 years of age in the same birth cohort, thus corrections were done for these in the current analysis [30, 31]. Birth weight was not adjusted in final regression model, based on the published literature from our site which concluded that birth weight was not a predictor for head circumference at 12 months [4]. Both bivariate as well as multivariate regression was performed and beta-co-efficients were expressed along with their 95% CI. Model fit was assessed using the adjusted R^2^ statistics. *P* value lesser than 0.05 was considered as statistically significant. We performed data analysis using Stata version 13 (StataCorp. 2013. Stata Statistical Software. Release 13. College station, TX: StataCorp LP).

## Results

After screening 301 pregnant mothers, 251 new-borns were recruited in the original birth-cohort. Excluded children were 50, including eight children who had a sibling already registered, seven had medical comorbidities, one was a multiple pregnancy, five families had pre-existing plan to migrate, nine mothers were not available for consent, ten families had combination of two or more of the above-mentioned reasons and ten mothers/parents refused participation. Subsequent 2-year and 5-year follow ups had 228 children and 212 children respectively. The loss to follow-up was mainly due to migration as illustrated in other articles, published from the same birth-cohort [30].

The Vellore birth-cohort had more than 80% of its families earning monthly income less than 1500 Indian rupees (20 USD) and 17% of babies weighed less than 2.5 kg at birth. The median (range) raw score of maternal cognition was noted as 46.5 (8–68) in the cohort. The cohort had girl predominance at birth (55%). Cohort characteristics at two and five years were similar with respect to sex distribution and SES characteristics (Table 1). Compared to birth, more children became stunted, malnourished and wasted by two years of age and this change was significant (Table 1).

**Table 1:** Comparison of cohort characteristics during enrolment, year 2 and 5 of follow-up of MAL-ED cohort

	Enrolment (***n*** = 251)	2 year (***n*** = 228)	5 year (***n*** = 212)	***P*** value^b^
**Gender**				0.960
Male	113 (45)	105 (46)	98 (46.2)	
Female	138 (55)	123 (54)	114 (53.8)	
**SES**				0.977
Low (WAMI <33rd percentile)	71 (30.2)	71 (31.14)	65 (30.66)	
High (WAMI ≥33rd percentile)	164 (69.79)	157 (68.86)	147 (69.34)	
**Height-for-age Z scores (HAZ)**^a^				**< 0.0001**
> − 2 SD	210 (83.67)	126 (55.51)	150 (70.75)	
< − 2 to ≥ − 3 SD	31 (12.35)	69 (30.40)	50 (23.58)	
< −3 SD	10 (3.98)	32 (14.10)	12 (5.66)	
**Weight-for-age Z scores (HAZ)**^a^				**0.020**
> − 2 SD	194 (77.29)	146 (64.32)	149 (70.28)	
< − 2 to ≥ − 3 SD	41 (16.33)	61 (26.87)	52 (24.53)	
< −3 SD	16 (6.37)	20 (8.81)	11 (5.19)	
**Weight-for-height Z scores**^a^				
> − 2 SD	203 (80.88)	202 (89)	179 (84.43)	**0.008**
< − 2 to ≥ − 3 SD	37 (14.74)	24 (10.57)	31 (14.62)	
< −3 SD	11 (4.38)	1 (0.44)	2 (0.94)	

^a^ Only 227 children had information on WAZ, HAZ and weight-for-length scores at 2 years of follow-up

^b^ Test of significance was done using Chi-squared statistics

At birth, around one-third of children had HC < − 2 SD, with this number increasing to 50% by 12 months. By 24 months, around 42.73% had HC between − 2 and − 3 SD and 8.81% of children had HC < − 3SD. The mean (SD) HC at 24 months was 44.9 (1.2) cm. The mean (SD) BSID cognition raw score at two years was 59.5 (3.4). The WPPSI mean (SD) raw scores at 5 years for verbal, performance and processing speed were 37.97 (9.2), 48.18 (9.8) and 35.84 (18.4) respectively.

For cognitive development at two years of age, HC < − 3 SD was a significant predictor with an adjusted beta co-efficient of − 2.21 (Table 2). The other factors included the mean blood lead levels and higher SES status. The R^2^ for the model was 7.6%.

**Table 2:** Predictors of cognitive development at 2 years of life in children of MAL-ED cohort

	Univariate analysis (***n*** = 227)		Multivariable analysis (***n*** = 226)	
Predictors	Unadjusted beta co-efficient^d^	P value	Adjusted beta co-efficient^d^	P value
**Cognition domain**^a^				
***Head circumference z-scores at 24 months***				
≥ −2 SD	ref	–	ref	–
< − 2 to ≥ −3 SD	**−**0.22 (−1.13–0.69)	0.633	− 0.16 (− 1.07–0.75)	0.730
< − 3 SD	**−2.57 (− 4.15 - -0.99)**	**0.002**	**− 2.21 (− 3.87 - -0.56)**	**0.009**
**Sex** (Female)	− 0.01 (− 0.90–0.87)	0.977	− 0.20 (− 1.09–0.69)	0.653
**Mean body iron**^**b**^	0.09 (− 0.03–0.20)	0.131	0.05 (− 0.06–0.17)	0.387
**Mean body lead**^c^	**−0.11 (− 0.19 - -0.03)**	**0.009**	**− 0.10 (− 0.18 - -0.02)**	**0.012**
***WAMI scores***				
< 33rd percentile	ref	–	ref	–
≥ 33rd percentile	**1.49 (0.55–2.42)**	**0.002**	**1.25 (0.25–2.26)**	**0.015**
**Mother’s cognition**	0.03 (−0.02–0.07)	0.203	0.01 (− 0.04–0.05)	0.779
**Length for age z-scores at 24 months**	0.18 (− 0.29–0.63)	0.466	−0.14 (− 0.63–0.33)	0.551

^a^R^2^ for the model - 7.6%

^b^Mean body iron levels were the average of body iron measurements at 7, 15 and 24 months of age in the cohort

^c^ Mean blood lead levels were the average of blood lead levels measured at 15 and 24 months of age in the cohort

^d^ Beta co-efficient is presented along with 95% confidence interval

For cognitive assessment at five years of age, R^2^ of models for verbal, performance and processing speed were 14.2, 15.5 and 17% respectively. HC < − 3 SD at two years of age predicted verbal (adjusted beta co-efficient of − 7.35) and performance (adjusted beta co-efficient of − 7.07) domains of cognition at five year; but not the processing speed (Table 3). The length for age z-scores at two years predicted processing speed at five years of age. Mean body iron levels and mother’s cognition also was significantly associated with verbal, performance and processing speed of five-year cognition.

**Table 3:** Predictors of cognition at 5 years of life in children of MAL-ED cohort

	Univariate analysis (***n*** = 212)		Multivariable analysis (n = 212)	
Predictors	Unadjusted beta co-efficient^d^	P value	Adjusted beta co-efficient^d^	P value
**Verbal domain**^a^				
***Head circumference z-scores at 24 months***				
> − 2 SD	ref	–	ref	–
< − 2 to ≥ − 3 SD	− 1.97 (− 4.49–0.56)	0.126	−1.58 (− 4.06–0.90)	0.211
< − 3 SD	**− 9.08 (− 13.44 - -4.71)**	**< 0.001**	**− 7.35 (− 11.78 - -2.92)**	**0.001**
**Sex** (Female)	1.12 (− 1.38–3.61)	0.379	−0.26 (− 2.69–2.17)	0.835
**Mean body iron**^**b**^	**0.54 (0.22–0.86)**	**0.001**	**0.42 (0.10–0.74)**	**0.010**
**Mean body lead**^c^	−0.10 (− 0.34–0.14)	0.423	−0.07 (− 0.30–0.16)	0.535
***WAMI scores***				
< 33rd percentile	ref	–	ref	–
≥ 33rd percentile	**3.06 (0.38–5.75)**	**0.025**	0.01 (− 2.74–2.74)	0.998
**Mother’s cognition**	**0.25 (0.14–0.36)**	**< 0.001**	**0.20 (0.08–0.32)**	**0.001**
**Length for age z-scores at 24 months**	**1.35 (0.08–2.63)**	**0.037**	0.45 (− 0.85–1.75)	0.493
**Performance domain**^a^				
***Head circumference z-scores at 24 months***				
> − 2 SD	ref	–	ref	–
< − 2 to ≥ − 3 SD	−1.79 (− 4.50–0.92)	0.194	−1.36 (− 3.99–1.28)	0.311
< − 3 SD	**− 9.29 (− 14 - -4.6)**	**< 0.001**	**−7.07 (− 11.77 - -2.36)**	**0.003**
**Sex**	−0.32 (− 2.99–2.35)	0.813	−1.91 (− 4.49–0.67)	0.145
**Mean body iron**^b^	**0.62 (0.28–0.97)**	**< 0.001**	**0.51 (0.17–0.85)**	**0.003**
**Mean body lead**^c^	−0.24 (− 0.50–0.02)	0.071	−0.22 (− 0.46–0.02)	0.068
***WAMI scores***				
< 33rd percentile	ref	–	ref	–
≥ 33rd percentile	**4.97 (2.15–7.80)**	**0.001**	2.27 (− 0.64–5.18)	0.125
**Mother’s cognition**	**0.23 (0.11–0.36)**	**< 0.001**	**0.16 (0.03–0.28)**	**0.014**
**Length for age z-scores at 24 months**	1.22 (− 0.14–2.59)	0.079	0.35 (−1.03–1.73)	0.617
**Processing speed domain**^a^				
***Head circumference z-scores at 24 months***				
> − 2 SD	ref		ref	
< −2 to ≥ − 3 SD	−1.15 (−6.31–4.00)	0.660	1.00 (− 3.90–5.89)	0.688
< − 3 SD	**− 14.09 (− 23.19 - -5.00)**	**0.003**	**−**7.47 (− 16.39 - -1.46)	0.101
**Sex**	**6.51 (1.56–11.47)**	**0.010**	3.15 (−1.65–7.95)	0.197
**Mean body iron**^b^	**1.25 (0.61–1.90)**	**< 0.001**	**0.94 (0.30–1.56)**	**0.004**
**Mean body lead**^c^	−0.16 (− 0.67–0.35)	0.540	−0.08 (− 0.55–0.38)	0.727
***WAMI scores***				
< 33rd percentile	ref	–	ref	–
≥ 33rd percentile	**8.67 (3.35–14.00)**	**0.002**	2.70 (−2.72–8.13)	0.327
**Mother’s cognition**	**0.47 (0.25–0.70)**	**< 0.001**	**0.31 (0.08–0.55)**	**0.009**
**Length for age z-scores at 24 months**	**4.97 (2.48–7.47)**	**< 0.001**	**3.48 (0.92–6.04)**	**0.008**

^a^R^2^ value for verbal, performance and processing speed domains are 14.2, 15.5 and 17.0% respectively

^b^Mean body iron levels were considered for the analysis and this is an average of body iron measurements at 7, 15 and 24 months of age in the cohort

^c^ Mean blood lead levels were considered for the analysis and this is an average of blood lead levels measured at 15 and 24 months of age in the cohort

^d^ Beta co-efficient is presented along with 95% confidence interval

## Discussion

This longitudinal prospective birth-cohort follow-up study from urban Vellore evaluated the effect of small HC on concurrently two-year cognition and predictively five-year cognition. HC < − 3 SD at two years of age was negatively associated with two-year cognition and predicted verbal and performance domains of five-year cognition, even after correction with SES, maternal cognition, mean iron and lead levels and length z-scores.

Two years is considered the culmination of the first 1000 days of life, where rapid brain growth and development makes this period a critical window for future individual potential [18]. The next 1000 days of life till five years of age is also crucial as child continues to learn and develop during this period [19]. The 2-year HC can reflect the brain volume more accurately than in later years, as fontanelles and sutures are open during this period [5, 6].

Scattered studies have recommended that routine HC measurement is unnecessary in typically developing (TD) children, but necessary for children at risk [5, 7, 8]. A longitudinal study done in south west United Kingdom showed that children with small HC (< −2SD) had increased risks of having lower intelligence at 8 years of age and associated neurodevelopmental disorder; but 85% of children with small HC did not have any disorder [5]. Routine HC measurements in Norwegian children were found useful in detecting hydrocephalus and cysts, when HC > +2SD [8]. A review analysis corroborated the above evidence [7]. However, this suggestion is in discordance with the AAP (http://brightfutures.aap.org) recommendation of measuring and plotting HC eight times in the first 2 years of age [1]. It must be noted that HC measurements in TD birth-cohorts have shown concurrent and predictive association with cognitive abilities. Analysis of the complete MAL-ED cohort from different LMIC countries, had shown early childhood HC growth patterns were associated with cognitive development at two years of age [2]. An Indian cohort study showed HC at birth was related to learning, memory and storage, and visuospatial abilities around 10 years of age [13]. Similarly HC at 2 years was associated with intelligence at both 4 and 8 years in a Bristol-based birth-cohort in the UK [14]. Reduced early childhood HC growth velocity had an increased risk of psychomotor delays in infancy as evidenced in both Israel [15] and Uruguay [16]. The current analysis adds further evidence to existing literature and concurs with AAP recommendation of HC measurement during routine immunisation visits. The Indian Academy of Pediatrics also recommends HC monitoring during immunisation visits till three years of age [32].

There is additional evidence to monitor HC routinely in children at risk, especially those born preterm, with low birth weight and those with antenatal teratogen exposure [9–12, 33, 34]. Brain volume, HC and cognition were found to be associated in a cross-sectional study of children with prenatal alcohol exposure in Canada [9]. In children born preterm, postnatal HC growth has been associated with developmental progress as evidenced in studies from Canada [10], Unites states [33], and Austria [34]. Among extremely low gestational age new-borns, microcephaly (HC < − 2 SD) at two years was concurrently associated with both motor and cognitive impairments [33].

Most of the above studies have used the definition of HC < − 2 SD to evaluate associated developmental risk. Microcephaly has had differing definitions of HC < − 2 SD [35] and < − 3 SD [36]. Many researchers have used the term ‘severe microcephaly’ for HC < −3SD [35]. The current cohort had almost one third of its children having HC < -2 SD at birth, with this number climbing to 50% by one year of age. A detailed HC evaluation from this cohort has been published and showed that maternal and paternal HC predicted one-year infant HC [4]. Even in the current cohort of proportionately higher individuals with small HC, microcephaly of HC < −3SD was associated concurrently and predictively with cognition at two and five years of age respectively. It might not be prudent to discard routine HC measurement in early childhood in general/paediatric practice, based on studies reporting microcephaly as HC < -2SD. Future community based studies can explore further if HC < -3 SD is a more appropriate definition of microcephaly.

Children in the present study from a LMIC setting were exposed to multiple environmental risks and had high blood lead levels and low serum iron levels in early childhood [30, 37], necessitating close developmental monitoring. Many children in similar LMIC settings across the world are exposed to multiple risks in early childhood itself. Routine developmental assessments for all children in the community level in LMIC settings are difficult, time-consuming and resource-draining. In such settings, HC measurements can be quick, reproducible, easy, and utilise a simple tool such as a measuring tape. Routine HC measurements at immunisation/community clinics can identify children with abnormal HC. This group at risk can be more closely monitored and supported with additional community-based neurodevelopmental aids to optimise their developmental potential.

There were limitations for the current study. Although the cohort follow-up was about 90 and 85% at two and five years respectively, the sample size of the cohort was relatively smaller. HC measurements can be subjective, but periodic re-training and checks as done in this study can help to overcome this deficiency. The current study has many strengths including systematic follow-ups of a LMIC birth-cohort, robust data granularity, standardized anthropometric and cognitive assessments, haematological analysis from a national reference-level laboratory and good quality assurance/control measures.

## Conclusion

Despite limitations, the current analysis done in a birth-cohort follow-up study in a LMIC setting showed that HC < -3SD at 2 years of age was associated concurrently with 2-year cognition and predictively with 5-year cognition; despite correction with SES, maternal cognition, early childhood iron and lead levels and length z-scores. This study highlights the utility of measuring early childhood HC in LMIC settings. Though there are differing definitions of microcephaly, HC < -3SD in typically developing populations can be defined as microcephaly, as these children are at risk of cognitive deficits. In resource poor settings, further studies need to confirm that microcephaly (HC < -3SD) can be used as an early risk marker of child development and cognition; and advance current evidence with appropriate neurodevelopmental interventions in this vulnerable group.

## Supplementary Information

**Additional file 1: Supplementary Table 1**. Multivariate analysis evaluating association of head circumference < − 2 SD and cognition in children of MAL-ED cohort.

## Data Availability

MAL-ED data from all sites are deposited in the https://clinepidb.org.website.

## Abbreviations

BSID-III: Bayley Scales of Infant and Toddler Development-III
HC: Head circumference
LMIC: Low and middle income countries
MAL-ED: The Etiology, Risk Factors and Interactions of Enteric Infections and Malnutrition and the Consequences for Child Health and Development
SES: Socio-economic status
WAMI: Water and sanitation, assets, maternal education and total household income
WPPSI-III: Wechsler Preschool Primary Scales of Intelligence – third edition
