# Supplementary material for: Association between head circumference at two years and second and fifth year cognition
Source: BMC Pediatr. 2021 Feb 11;21:74. doi: 10.1186/s12887-021-02543-0 (PMC7876785; doi:10.1186/s12887-021-02543-0)
Supplement: Supplementary file 1 — Additional file 1: Supplementary Table 1. Multivariate analysis evaluating association of head circumference < − 2 SD and cognition in children of MAL-ED cohort. [file 12887_2021_2543_MOESM1_ESM.docx]

**Association between head circumference at two years and second and fifth year cognition**

Title page

**Manuscript title**

Association between head circumference at two years and second and fifth year cognition

**Authors**

Beena Koshy^1^, Manikandan Srinivasan^2^, Timiri Palani Murugan^1^, Anuradha Bose^3^, Pamela Christudoss^4^, Venkata Raghava Mohan^3^, Sushil John^5^, Reeba Roshan^1^, Gagandeep Kang^2^

^1^ - Developmental Paediatrics Unit, Christian Medical College, Vellore

^2^ - Wellcome research Unit, Christian Medical College, Vellore

^3^ -Community Health, Christian Medical College, Vellore

^4^ –Clinical Biochemistry, Christian Medical College, Vellore

^5^ -Low Cost Effective Care Unit, Christian Medical College, Vellore

**Author list**

Dr. Beena Koshy, MD (Paediatrics), MD (Res), Developmental Paediatrics Unit,

Christian Medical College,Vellore-632004. [beenakurien@cmcvellore.ac.in](mailto:beenakurien@cmcvellore.ac.in)

Dr. Manikandan Srinivasan, MD (Community Health), Wellcome research Unit,

Christian Medical College,Vellore-632004.[manikandanmbbs06@gmail.com](mailto:manikandanmbbs06@gmail.com)

Dr. Timiri Palani Murugan, MD (Paediatrics), Developmental Paediatrics Unit,

Christian Medical College,Vellore-632004. [murugantp007@gmail.com](mailto:murugantp007@gmail.com)

Dr. Anuradha Bose, MD (Paediatrics), Community Health

Christian Medical College,Vellore-632004 [anubose99@gmail.com](mailto:anubose99@gmail.com)

Dr. Pamela Christudoss, MD (Biochemistry), Clinical Biochemistry

Christian Medical College,Vellore-632004 [pamela@cmcvellore.ac.in](mailto:pamela@cmcvellore.ac.in)

Dr. Venkata Raghava Mohan, MD (Community Health), Community Health

Christian Medical College,Vellore-632 004. [venkat@cmcvellore.ac.in](mailto:venkat@cmcvellore.ac.in)

Dr. Sushil John, MD (Community Health), Low Cost Effective Care Unit,

Christian Medical College,Vellore-632 004. [rikkisush@cmcvellore.ac.in](mailto:rikkisush@cmcvellore.ac.in)

Dr. Reeba Roshan, PhD, Developmental Paediatrics Unit,

Christian Medical College,Vellore-632 004. [reebaroshan@gmail.com](mailto:reebaroshan@gmail.com)

Dr. Gagandeep Kang, MD (Microbiology), PhD, Wellcome Research Unit.

Christian Medical College,Vellore-632 004. [gkang@cmcvellore.ac.in](mailto:gkang@cmcvellore.ac.in)

**Corresponding author**

Dr. Beena Koshy, MD (Paed), MD (Res, UK), PDFDP

Professor

Developmental Paediatrics

Christian Medical College, Vellore

beenakurien@cmcvellore.ac.in

Ph: 914162283260

**Running Head**

Head circumference and cognition in early childhood

**Supplementary table 1: Multivariate analysis evaluating association of head circumference < -2 SD and cognition in children of MAL-ED cohort**

|  | **Cognition at 2 years (n=226)** | **Cognition at 5 years (n=212)** | | |
| --- | --- | --- | --- | --- |
| ***Predictors*** | Cognitive development | Verbal domain | Performance domain | Processing speed domain* |
|  | ***Adjusted beta co-efficients with 95% CI*** | | | |
| ***Head circumference z-scores at 24 months*** | | | | |
| > -2 SD | ref | ref | ref | ref |
| < -2 SD | -0.46 (-1.36 – 0.44) | -2.41 (-4.84 – 0.03) | -2.18 (-4.75 – 0.40) | -0.17 (-4.95 – 4.61) |
| **Sex** (Female) | -0.28 (-1.19 – 0.62) | -0.47 (-2.93 – 1.98) | -2.12 (-4.73 – 0.48) | 2.80 (-2.02 – 7.61) |
| **Mean body iron**^#^ | 0.07 (-0.05 – 0.19) | **0.45 (0.13 – 0.78)** | **0.54 (0.20 – 0.88)** | **0.98 (0.34 – 1.61)** |
| **Mean body lead^$^** | **-0.09 (-0.17 – 0)** | -0.08 (-0.31 – 0.15) | -0.23 (-0.48 – 0.01) | -0.09 (-0.56 – 0.38) |
| ***WAMI scores*** | | | | |
| < 33^rd^ percentile | ref | ref | ref | ref |
| ≥ 33^rd^ percentile | **1.34 (0.32 – 2.36)** | 0.21 (-2.56 – 2.99) | 2.49 (-0.45 – 5.42) | 3.10 (-2.34 – 8.54) |
| **Mother’s cognition** | 0.01 (-0.04 – 0.05) | **0.21 (0.09 – 0.32)** | **0.16 (0.04 – 0.29)** | **0.32 (0.09 – 0.55)** |
| **Length for age z-scores at 24 months** | -0.03 (-0.51 – 0.45) | 0.77 (-0.52 – 2.06) | 0.66 (-0.70 – 2.03) | **3.91 (1.38 – 6.45)** |

*N=210

^#^Mean body iron levels were considered for the analysis and this is an average of body iron measurements at 7, 15 and 24 months of age in the cohort

^$^ Mean blood lead levels were considered for the analysis and this is an average of blood lead levels measured at 15 and 24 months of age in the cohort
